# Evolution of MET and NRAS gene amplification as acquired resistance mechanisms in EGFR mutant NSCLC

**DOI:** 10.1038/s41698-021-00231-x

**Published:** 2021-10-12

**Authors:** T. L. Peters, T. Patil, A. T. Le, K. D. Davies, P. M. Brzeskiewicz, H. Nijmeh, L. Bao, D. R. Camidge, D. L. Aisner, R. C. Doebele

**Affiliations:** 1 Department of Medicine, Division of Medical Oncology, University of Colorado- Anschutz Medical Campus, Aurora, CO USA; 2 Department of Pathology, University of Colorado- Anschutz Medical Campus, Aurora, CO USA

**Keywords:** Molecular medicine, Targeted therapies, Cancer therapeutic resistance, Non-small-cell lung cancer

## Abstract

*EGFR* mutant non-small cell lung cancer patients' disease demonstrates remarkable responses to EGFR-targeted therapy, but inevitably they succumb to acquired resistance, which can be complex and difficult to treat. Analyzing acquired resistance through broad molecular testing is crucial to understanding the resistance mechanisms and developing new treatment options. We performed diverse clinical testing on a patient with successive stages of acquired resistance, first to an EGFR inhibitor with *MET* gene amplification and then subsequently to a combination EGFR and MET targeted therapies. A patient-derived cell line obtained at the time of disease progression was used to identify *NRAS* gene amplification as an additional driver of drug resistance to combination EGFR/MET therapies. Analysis of downstream signaling revealed extracellular signal-related kinase activation that could only be eliminated by trametinib treatment, while Akt activation could be modulated by various combinations of MET, EGFR, and PI3K inhibitors. The combination of an EGFR inhibitor with a MEK inhibitor was identified as a possible treatment option to overcome drug resistance related to *NRAS* gene amplification.

## Introduction

Lung cancer is the most frequently diagnosed cancer in both men and women in the United States, and the leading cause of cancer-related deaths^1^. The histological subset of lung cancers termed non-small cell lung cancer (NSCLC) has benefitted greatly from the era of precision medicine, where numerous molecular drivers, such as mutant epidermal growth factor receptor (*EGFR*) or BRaf proto-oncogene (*BRAF*), can now be identified in a majority of patients and they can be given drugs that directly target those drivers^2^. Patients with EGFR-driven NSCLC respond favorably to EGFR-targeted therapies, with longer survival and fewer side effects compared with chemotherapy^3–6^. However, tumors inevitably become resistant to these therapies. Acquired resistance can be due to secondary mutations in the target (EGFR) that render the therapy ineffective, activation of bypass signaling pathways to maintain tumor growth, or cellular transformation^7^. Amplification of *MET* was the first example of resistance to EGFR therapy through bypass signaling identified, where MET activated phosphoinositide 3-kinase (PI3K) signaling through EGFR family member ERBB3^8^. Since then MET has been confirmed as a bona fide resistance mechanism in EGFR and other oncogene-driven NSCLC^9,10^. Activation of the mitogen-activated protein kinase (MAPK) signaling pathway through mutations in BRaf proto-oncogene (BRAF) was also found in a small percentage of samples resistant to EGFR therapy^11^. While next-generation inhibitors have been developed to target common resistance mutations, such as osimertinib for EGFR T790M, acquired resistance due to bypass signaling is more difficult to detect by either tumor or circulating tumor DNA (ctDNA) testing and, even when detected, to treat succesfully^12,13^. Here we report a case of successive stages of bypass-mediated acquired resistance to erlotinib and crizotinib therapy, culminating in cancer with three different identifiable drivers including EGFR, MET, and NRAS.

## Results

### Patient case report

**Fig. 1:**
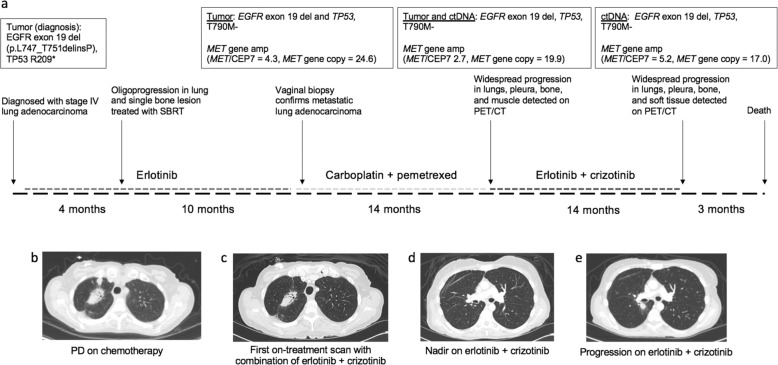
Patient treatment history. **a** Timeline of the patient’s diagnosis and treatments, with molecular testing results shown in boxes. **b** CT scan demonstrates post chemotherapy (pre-erlotinib/crizotinib) with lung nodules. **c** The first on-treatment CT chest scan during erlotinib and crizotinib treatment demonstrate reduction or disappearance of lung nodules. **d** A CT chest of this patient at the radiographic nadir while on erlotinib and crizotinib with complete resolution of lung nodules. **e** A CT chest of this patient at the time of progression on erlotinib and crizotinib with new perihilar lung nodules.

A 57-year-old Caucasian woman with a remote 5-pack-year smoking history presented with a cough. Ultimately, a computed tomography (CT) scan of the chest demonstrated a large right upper lobe mass. A bronchoscopy along with transbronchial biopsies of the right upper lobe mass and lymph nodes all demonstrated poorly differentiated lung adenocarcinoma. Positron emission tomography (PET) re-identified a highly FDG-avid right upper lobe lung mass along with multiple osseous metastases. Magnetic resonance imaging of the brain revealed multiple enhancing lesions. Initial molecular testing using Sanger sequencing identified an EGFR Exon 19 deletion (p.747_S752delinsP). Prior to initiating first-line systemic therapy, the patient received whole-brain radiotherapy. The patient was started on erlotinib 150 mg daily, but required a dose reduction to 100 mg owing to the development of acneiform rash (Fig. 1a). The patient developed oligoprogression in the bone and lung approximately four months after starting erlotinib, for which she received stereotactic body radiotherapy.

**Fig. 2:**
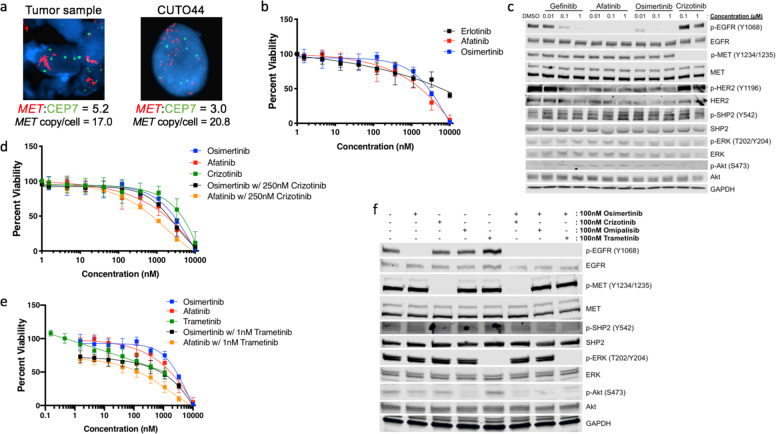
CUTO44 cell line is resistant to single-agent EGFR inhibition but sensitive to combination with crizotinib or trametinib. **a** MET FISH analysis of the patient tumor sample upon progression on erlotinib (left) and the CUTO44 cell line (derived from the same biopsy, right) showing MET probes labeled in red and the chromosome 7 centromere probes in green. **b** Cell proliferation inhibition following treatment with erlotinib, afatinib, or osimertinib. CUTO44 cells were treated with the indicated concentration of drugs for 72 h and proliferation was measured by MTS assay. Showing the mean ± SD, *n* = 3 biological replicates. **c** Western blot showing downstream signaling changes following treatment with EGFR inhibitors and crizotinib. CUTO44 cells were treated with the indicated drugs for 2 h before lysis and protein analysis by western blot. Showing representative images, *n* = 2. **d** Cell proliferation following treatment with crizotinib and the combination with EGFR inhibitors. Same as in **b**. **e** Cell proliferation inhibition of cells treated with trametinib and the combination with EGFR inhibitors. Same as in **b**. **f** Western blot showing downstream signaling changes following drug combination treatments. Same as in **c** with drugs combined at the indicated doses.

Approximately 10 months after starting erlotinib, the patient developed abnormal uterine bleeding and vaginal pain, leading to a vaginal wall biopsy that showed poorly differentiated carcinoma, consistent with metastasis from her lung adenocarcinoma. Although molecular testing was pending, the patient-initiated carboplatin (AUC 6) plus pemetrexed 500 mg/m^2^ for six cycles followed by maintenance pemetrexed. Molecular testing using a multi-gene next-generation sequencing (NGS) assay and fluorescent in situ hybridization (FISH) revealed *MET* gene amplification (*MET*/CEP7 ratio = 4.3, *MET* gene copy number = 24.6) and a *TP53* p.R209* mutation (Figs. 1a, 2a). The patient developed widespread metastatic progression in lungs, pleura, bone, and soft tissue after 15 cycles of maintenance pemetrexed (Fig. 1b). Molecular testing on a progressive lung lesion and ctDNA sample both identified a *TP53* p.R209* and *NF1* p.V2649M mutation. *MET* amplification was re-identified by FISH, with a *MET/*CEP7 ratio of 2.7 and gene copy number of 19.9. The patient next received a combination of erlotinib and crizotinib with an initial partial response to therapy (Fig. 1c, d). She remained on this combination therapy for ~14 months before developing progression in the lung and brain. A repeat lung biopsy demonstrated persistent *MET* amplification (*MET*/CEP7 ratio = 5.2, *MET* gene copy number = 17.0) and a cell line was obtained (Fig. 1e). The patient was screened for multiple clinical trials. However, the patient developed rapidly progressive disease and died.

### Cell line characterization

At the time of the biopsy following progression on erlotinib plus crizotinib, a portion of the tumor biopsy sample was collected for cell line derivation. The cell line that grew out from the sample was termed CUTO44 and maintained *MET* gene amplification with a *MET*/CEP7 ratio of 3.0 and *MET* gene copy number of 20.8, and demonstrated resistance to single-agent first (erlotinib), second (afatinib), and third (osimertinib) generation EGFR inhibitors in cell viability assays, with half-maximal inhibitory concentrations (IC_50_) >1700 nM for all single agents (Fig. 2a, b). Treatment of CUTO44 cells with gefitinib, afatinib, and osimertinib inhibited EGFR phosphorylation, consistent with a lack of kinase domain mutation such as EGFR p.T790M (Fig. 2c); however, downstream activation of the extracellular signal-related kinase (ERK) and the protein kinase B (Akt) signaling pathways were maintained, suggestive of a bypass signaling mechanism (Fig. 2c). We also observed high expression and phosphorylation of HER2, which was blocked by the pan-HER inhibitor afatinib, but could not detect the expression of HER3 (Fig. 2c, data not shown).

Next, we evaluated signaling following treatment with the MET inhibitor crizotinib, and found while it successfully blocked MET activation, it had no effect on downstream MAPK signaling (Fig. 2c). Crizotinib treatment did decrease phosphorylation of Akt, consistent with the role of MET signaling via the PI3K/Akt pathway^14,15^. The combination of a low dose of crizotinib with either osimertinib or afatinib resulted in a reduction of cell viability and was moderately synergistic, with a combination index of 0.818 and 0.515 for the combination of osimertinib or afatinib with crizotinib, respectively (Fig. 2d). Recently, inhibitors of the phosphatase SHP2 have been investigated to determine whether they can overcome resistance in oncogene-driven cancers because of the role of SHP2 in signal transduction from multiple receptor tyrosine kinases^16,17^. SHP2 phosphorylation was completely resistant to EGFR or MET inhibition (Fig. 2c). To determine whether SHP2 signaling might be driving resistance via another RTK, we tested whether two SHP2 inhibitors, SHP-099 and RMC4550, alone or when combined with osimertinib or afatinib restored the sensitivity of CUTO44, but they had no effect on cell proliferation (Supplementary Fig. 1a, b)^18^.

Maintenance of downstream signaling despite inhibition of EGFR and/or MET suggested there was another component driving MAPK activation (Fig. 2c). To determine first whether MAPK activation was responsible for resistance, we tested trametinib, a mitogen-activated protein kinase 1 and 2 (MEK1/2) inhibitor alone or in combination with the EGFR inhibitors osimertinib or afatinib (Fig. 2e). Trametinib alone inhibited viability only at high doses, but the combination of low dose trametinib with osimertinib or afatinib was highly synergistic, reducing the IC_50_ of osimertinib from 1722 to 60 and afatinib from 931 nM to 21 nM, with combination indices of 0.288 and 0.085, respectively. We confirmed these results with another MEK inhibitor, cobimetinib (Supplementary Fig. 1c). The lack of single-agent activity by either MEK inhibitor indicated that EGFR was still actively signaling either through the PI3K/Akt pathway or reactivating MAPK signaling at a later timepoint.

To better understand the complex resistance observed in this cell line, we next tried various combinations of targeted inhibitors and evaluated downstream signaling changes. Inhibition of MET with crizotinib had a modest effect on Akt activation, but combining osimertinib with crizotinib almost completely eliminated Akt activation (Fig. 2f). In addition, the PI3K inhibitor omipalisib completely eliminated Akt activation as a single agent or when combined with osimertinib, but had no effect ERK activation. Surprisingly, CUTO44 cells were relatively sensitive to omipalisib as a single agent, but more sensitive to the combination of EGFR and a MEK inhibitor (Supplementary Fig. 1d). Trametinib was able to completely eliminate MAPK activation alone and when combined with osimertinib. Taken together these data suggest that EGFR and MET were primarily driving Akt activation, whereas EGFR and an unknown component upstream of MEK were driving MAPK activation in the resistant cells.

### Identification of *NRAS* amplification by comparative genomic hybridization

**Fig. 3:**
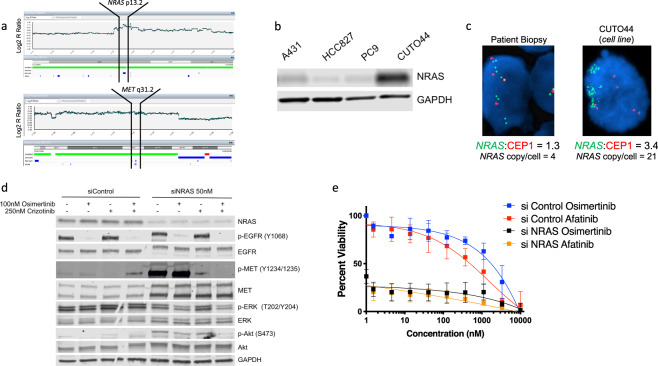
Identification of *NRAS* gene amplification by CGH and sensitivity to *NRAS* knockdown combined with EGFR inhibition. **a** *NRAS* and *MET* amplification identified by CGH. CUTO44 genomic DNA was analyzed by CGH using Illumina CytoSNP-850K bead array. Images show chromosomes 1 and 7, highlighting the regions containing *NRAS* and *MET*. **b** NRAS protein expression in EGFR mutant lung cancer cell lines. Western blot showing NRAS protein levels in EGFR mutant lung cancer cell lines. Representative images are shown. **c** *NRAS* FISH on the patient biopsy after progression on erlotinib (left) and in the CUTO44 cell line (right) with *NRAS* probe in green and chromosome 1 centromere probes in red. **d** Protein expression changes following *NRAS* knockdown by siRNA. CUTO44 cells were transfected with 50 nM *NRAS* siRNA for 48 h, then treated with the indicated drug combinations for 2 h prior to lysis for western blot analysis of protein expression. Representative images, *n* = 2. **e** Cell viability following *NRAS* knockdown. CUTO44 cells were transfected with *NRAS* siRNA for 48 h prior to being treated with the indicated concentration of drugs for 72 h and viability was measured by MTS assay. Showing the mean ± SD, *n* = 3 biological replicates.

We submitted the cell line for comparative genomic hybridization (CGH) to determine whether any copy number alterations could explain the combination resistance observed in the patient-derived cell line. The data revealed numerous copy number gains and losses across most chromosomes in the cell line, including evidence for chromothrypsis of chromosome 7, with the region containing the *MET* gene amplified, consistent with the clinical testing results (Fig. 3a). Notably, this focal gene amplification did not involve *BRAF*, which is located in proximity to *MET* on chromosome 7 (Supplementary Fig. 2, data not shown) Interestingly, there was also high-level amplification of the *NRAS* gene, with an R ratio equivalent to more than or equal to four copies of the gene (Fig. 3a). When we compared NRAS protein levels to other EGFR mutated NSCLC cell lines HCC827 and PC9, we found the CUTO44 cell line had markedly higher protein expression of NRAS (Fig. 3b).

We confirmed substantial *NRAS* gene amplification in the CUTO44 cell line using *NRAS* FISH probes and observed an average of 21 copies of *NRAS* per cell and an *NRAS*/CEP1 ratio of 3.4, and *NRAS* amplification was not present in the patient tumor sample biopsied prior to crizotinib therapy (Fig. 3c). To determine if *NRAS* amplification was present in the final patient biopsy at progression on erlotinib and crizotinib we compared the average reads from the NGS assay in the patient sample to the average reads from samples of normal donor blood. We found *NRAS* reads in the normal blood samples to be 0.93 ± 0.03, and 9.37 in the patient, suggesting that high-level *NRAS* amplification was present in the biopsy at progression on erlotinib and crizotinib. We also confirmed the previously identified *MET* amplification by this method, where MET reads in the normal blood samples were 1.02 ± 0.03 and 2.39 in the patient sample, suggesting that analysis of clinical NGS assays can accurately identify genomic amplifications. We retrospectively compared the averaged *NRAS* read counts from 21 other *EGFR* mutant patients who were treated at the University of Colorado Cancer Center between 2018 and 2020 and had the same clinical NGS assay data and found that none of the other patients had detectable *NRAS* amplification, with average read counts in the same range as normal controls (Supplementary Fig. 3a).

To confirm that amplified *NRAS* was the unknown component activating the MAPK pathway in the CUTO44 cells we used siRNA to evaluate downstream signaling in the control *NRAS* knockdown cells. We found that *NRAS* knockdown alone modestly decreased ERK phosphorylation, and when combined with osimertinib this could be decreased further, though not completely eliminated (Fig. 3d). *NRAS* knockdown alone or when combined with osimertinib had little effect on phosphorylation of Akt, but the triple combination with crizotinib was able to nearly eliminate its activity. The *NRAS* knockdown also dramatically sensitized the cells to either osimertinib or afatinib treatment, reducing the IC_50_’s of the either drug to subnanomolar concentrations (Fig. 3e and Supplementary Fig. 3b, c).

## Discussion

This case report demonstrates the success of sequential targeted therapies, as we were able to identify amplified *MET* as the initial resistance mechanism to EGFR inhibition and combine crizotinib with erlotinib to extend survival of the patient to ~4 years. MET was the first bypass signaling mechanism of resistance identified in EGFR and recently the TATTON trial demonstrated early efficacy and safety of the combination of osimertinib and the MET inhibitor savolitinib^8,19^.

However, this case also highlights the challenge of chasing tumor evolution with successive rounds of targeted therapy, which can select for increasingly complex and difficult to treat cancers. Interestingly, in this tumor, the dependence on the primary oncogene, EGFR, was maintained throughout the evolution of acquired resistance. No single-agent therapy we tested was effective against the primary cell line, and we only achieved synergistic inhibition of cell viability when we combined either crizotinib or trametinib with EGFR inhibition, demonstrating the maintenance of at least partial dependence on EGFR. Though *MET* amplification was fairly consistent through the course of the patient’s treatment and in the derived cell line, the dependence on MET signaling seemed to be more plastic. In the CUTO44 cell line, Akt activation was driven by MET and EGFR, but the combination of EGFR inhibitors with crizotinib was not as synergistic as the combination of an EGFR inhibitor with trametinib, or knockdown of *NRAS*. This indicated a shift from MET dependence to NRAS dependence, given the patient’s prior response to combination EGFR and MET inhibition. *MET* amplification was maintained over three years of repeated clinical biopsies and the generation of a patient-derived cell line with the high copy number only changing slightly, whereas the ratio with the chromosome 7 centromere contributed the majority of the variability to the amplification level in repeated biopsies.

There have been several reports of *NRAS* activating mutations following EGFR-targeted therapy in patients, but these studies do not always utilize assays that can reveal genomic amplification beyond a few genes such as *HER2* and *MET*^11,20^. The increasing use of ctDNA analysis at disease progression and lack of a standardized cutoff for *NRAS* or other oncogene amplification using FISH or NGS testing on tumor biopsy samples may contribute to the underrepresentation of gene amplification as an important bypass signaling resistance mechanism in cancers. *NRAS* mutations have also been identified as resistance mechanisms using patient-derived cell line models harboring *RET* and *ROS1* gene fusions, further supporting the idea that cancers often develop resistance using a somewhat finite group of oncogenes following TKI treatment^13,21,22^. Studies of in vitro induced EGFR resistance, however, have revealed that *NRAS* amplification can drive EGFR TKI resistance in cell lines, suggesting this could be a potential resistance mechanism in patients as well^23,24^. We identified the first patient to our knowledge with acquired resistance to erlotinib and crizotinib driven by *NRAS* amplification, but unfortunately, this determination was not made until after the patient died. We attempted to express NRAS in an alternate system to independently show its ability to drive resistance to EGFR and MET inhibitors, but EGFR models with MET overexpression are not readily available and we were not able to overexpress NRAS in an EGFR mutant cell line. This could be due to the toxicity of the overactivation of the MAPK pathway^21,25^.

We also extended our novel use of clinical NGS data to evaluate genomic amplifications to a small cohort of 21 *EGFR* mutant NSCLC patients. Though we did not identify any other patients with *NRAS* amplification, this analysis can be a useful tool for revealing acquired resistance driven by amplification of commonly sequenced genes. This case report corroborates the in vitro studies, suggesting *NRAS* amplification is a relevant resistance mechanism to EGFR-targeted therapy and should be evaluated at the time of EGFR TKI resistance, especially given our in vitro evidence for a potential rational combination therapy to treat such patients. Taken together, our data underscore the complexity and plasticity of acquired resistance to targeted therapies and the importance of using molecular assays to evaluate mutations as well as copy number alterations when trying to identify acquired resistance mechanisms. This work also highlights the critical importance of patient-derived cell lines which can be repeatedly tested using both pharmacologic and genetic manipulations as well as other techniques to identify novel resistance pathways. Finally, we identified a potential combination of EGFR and MEK-targeted therapies as a possible treatment option for patients with EGFR-driven lung cancer with *NRAS* gene amplification as a bypass signaling pathway.

## Methods

### Clinical testing

Molecular testing was conducted by the CLIA certified Colorado Genetics Laboratory (CGL) and Colorado Molecular Correlates Laboratory (CMOCO) and performed using laboratory assays that were independently validated. Mutational analyses for select targets were performed by using Sanger sequencing and allele-specific qPCR (RGQ platform, Qiagen, Hilden, Germany). For broader mutational analysis, a customized version of the ArcherDx VariantPlex Solid Tumor library preparation kit was used (ArcherDx, Boulder, CO). Libraries from this assay were sequenced on the Illumina platform, and raw sequence data were processed using the ArcherDx Analysis package v5.1.2. Libraries were sequenced on the Illumina platform, and raw sequence data were processed using the ArcherDx Analysis package v4.1.1.7. For ctDNA analysis, we used the Guardant360 NGS assay (Illumina, San Diego, CA) using hg19 as the reference genome. For all research performed using patient data in this study appropriate approval was obtained and all ethical regulations related to the use of this data were followed.

For the CUTO44 patient cell line, CGH DNA was extracted using the Promega RSC automated extraction procedure and Chromosomal microarray was processed on the Illumina® CytoSNP-850K bead array. The CytoSNP-850K array targets >250 genomic regions in accordance with the most recent ICCG recommendations. The array can detect copy number losses and gains with a resolution of ~10 kb. BlueFuse Multi v4.4 software was used to analyze the data and calls were made at 1 Mb for a loss and 2 Mb for a gain^26^. The Genome Reference Consortium Human genome Build 37 (hg19) is used in this study. Data are deposited in Gene Expression Omnibus under the accession number GSE180894.

*MET* FISH was done by the CGL using Vysis probes from Abbott Laboratories (Abbott Park, IL) and processed using CytoVison (Leica Biosystems, Buffalo Grove, IL). The NRAS FISH probe was purchased from Empire Genomics (Wiliamsville, NY). Amplification was considered as a gene to centromere ratio of >2.5 for both *MET* and *NRAS*.

To assess gene amplification in the final patient biopsy at progression on erlotinib and crizotinib, we averaged the Archer VariantPlex unique read counts from all primers to the gene (*MET*, *NRAS*, or *EGFR*) and normalized that average to the average number of unique reads originating from all primers in the assay. We then compared this value with averaged values from five blood samples from healthy individuals (which should represent the diploid state for the genes)^27^. EGFR patient sample data were generated under Colorado Multiple Institution Review Board (COMIRB)-approved protocol 09-0183, where a full waiver of consent was given by COMIRB owing to minimal risk to subjects. The normal blood samples data were derived from de-identified samples used by CMOCO for assay validation. *NRAS* read counts from *EGFR* mutant patients with Archer VariantPlex assay data between 2018 and 2020 treated at the University of Colorado Cancer Center with IRB approval and written informed consent.

#### Cell line derivation and cell culture and reagents

CUTO44 cell line derivation was performed as described previously, with patient consent obtained under COMIRB-approved protocol 11-1621 with written consent^28^. A431, PC9, HCC827, and 293 T cells were purchased from the University of Colorado Anschutz Medical Campus Cell Technologies Shared Resource. 293 T cells were cultured in DMEM media (ThermoFisher, 12430112) with 10% FBS (ThermoFisher) and all other cell lines were cultured in RPMI1640 (ThermoFisher, 21875034) with 10% FBS (ThermoFisher, A4766) at 37° with 5% CO_2_.

#### MTS assay

CUTO44 cells were seeded at the appropriate density (determined empirically) in 96-well plates and treated with the indicated concentration of drug the following day. After 72 h MTS reagent was added per manufacturers recommendations (Promega, G7570) and absorbance was measured on a microplate reader (BioTek). IC_50_ values were calculated using non-linear regression curve fitting in GraphPad Prism. The combination index was calculated based on the IC_50_ using the Chou-Talay method^29^. Osimertinib, afatinib, gefitinib, trametinib, SHP-099, RMC4550, crizotinib, cobimetinib, and omipalisib were purchased from Selleck Chemicals (S7297, S1011, S1025, S2673, S8278, S8718, S1068, S8041, S2658).

#### Western blot

Western blot was performed as described previously^28^. All drug treatments were done at the indicated doses for 2 hours. All blots were derived from the same experiment and processed in parallel. Antibodies used were p-EGFR Y1068 (377 S), MET (3148 S), p-MET Y1234/1235 (3077 S), p-HER2 Y1196 (6942 S), p-SHP2 Y542 (3751 S), ERK1/2 (4696), p-ERK1/2 T202/Y204 (4370), Akt (2920), p-Akt S473 (4060) from Cell Signaling Technology; EGFR (610017), HER2 (610162), and SHP2 (610622) from BD Biosciences; GAPDH (MAB374) from Millipore; and NRas (SCF155) from Santa Cruz Biotechnology. All cell signaling and BD Biosciences antibodies were diluted 1:1000, the GAPDH antibody was diluted 1:5000 and the NRAS antibody was diluted 1:200.

#### NRas knockdown

In all, 50 nM NRAS siRNA (Horizon, L-003919-00-00035) was transfected into the CUTO44 cells using FuGene HD transfection reagent (Promega, E2311), per manufacturers protocol. The following day cells were plated for MTS assay and protein analysis, which was done 48 h after transfection.

#### NRas knockdown cell line generation

Lentiviral particles were generated by transfecting 293 T cells with control or NRAS shRNA plasmids along with the second generation lentiviral packaging, purchased from the University of Colorado Functional Genomics Core (Sigma Mission, SHCLND-NM_010937 and SHP001), using the TransiIT-293 reagent (Mirus Bio, MIR 2704). Target cells were transduced with the viral particles and selected in puromycin prior to downstream analysis.

### Reporting summary

Further information on research design is available in the Nature Research Reporting Summary linked to this article.

## Supplementary information

Reporting Summary

Supplementary Information

## Data Availability

All the data and resources generated for this study are available in the article or from the corresponding author upon request. CGH array data are deposited in GEO under the accession number GSE180894.
