# Supplementary material for: Evolution of MET and NRAS gene amplification as acquired resistance mechanisms in EGFR mutant NSCLC
Source: NPJ Precis Oncol. 2021 Oct 12;5:91. doi: 10.1038/s41698-021-00231-x (PMC8511249; doi:10.1038/s41698-021-00231-x)

Supplementary Figure 1

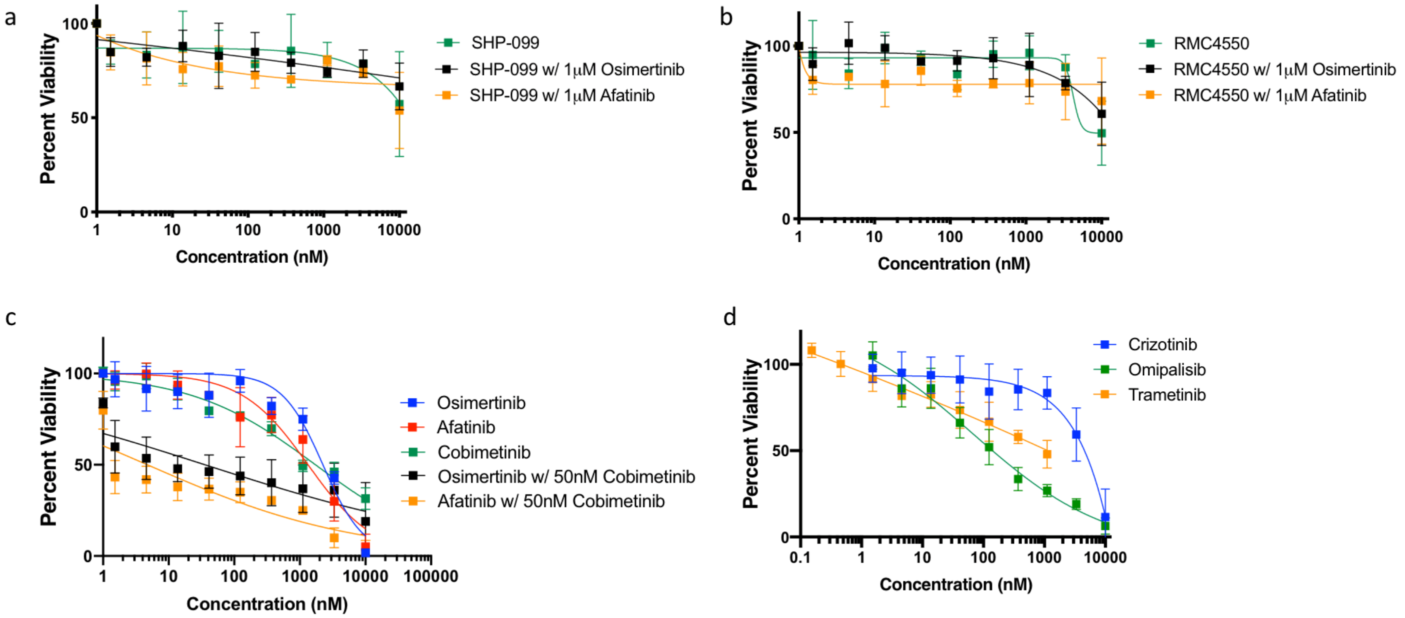

Supplementary Figure 1. CUTO44 cell line is resistant to EGFR inhibitor combinations with SHP2 inhibitors or other pathway inhibitors. Cell viability to either SHP-099 (**a**) or RMC4550 (**b**) alone or combined with EGFR inhibitors. CUTO44 cells were treated with the indicated concentration of drugs for 72 hours and proliferation was measured by MTS assay. Showing the mean+/- SD, n=3 biological replicates. **c** Cell proliferation inhibition of cells treated with cobimetinib alone and in combination with EGFR inhibitors. Same as in a. **d** Single agent crizotinib, omipalisib or trametinib. Same as in a.

Supplementary Figure 2

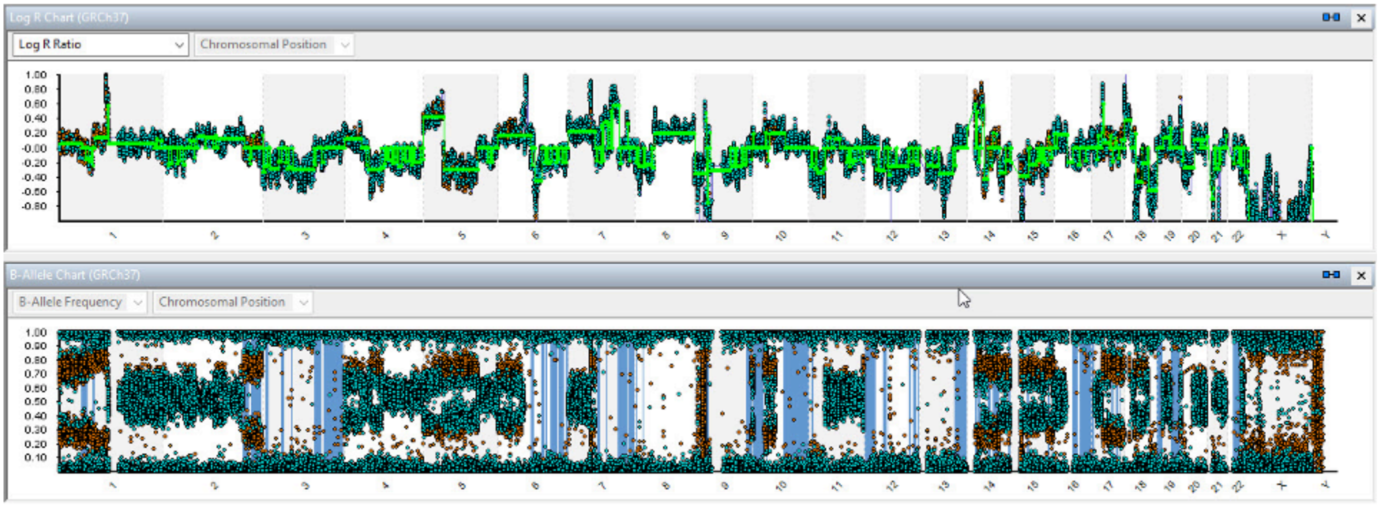

Supplementary Figure 2. Whole genome view of the CUTO44 CGH microarray analysis.

Supplementary Figure 3

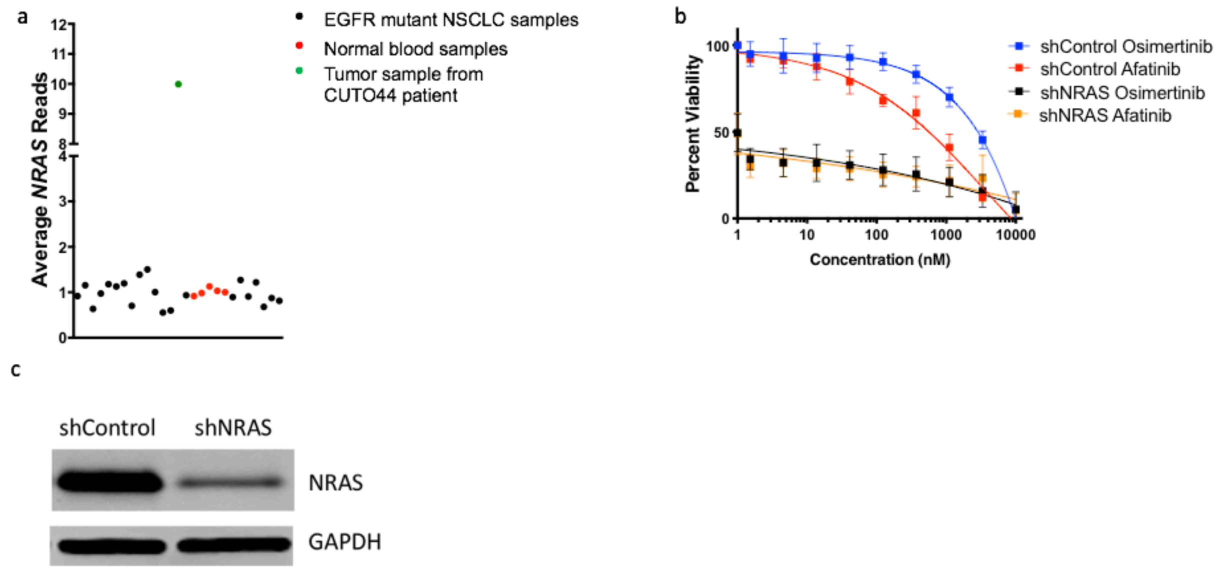

Supplementary Figure 3. NRAS knockdown sensitizes CUTO44 cells to EGFR inhibitors. **a** Average, normalized *NRAS* read counts from the Archer VariantPlex assay in a cohort of EGFR mutant NSCLC patients with progression on EGFR inhibitors. Average read counts from five different normal patient blood samples shown as a control in red. **b** Cell viability following NRAS knockdown in stable cell lines. Control and NRAS shRNA knockdown cell lines were generated by lentiviral transduction then sensitivity to EGFR inhibitors was measured after 72 hours of drug treatment using MTS assays. Showing the mean+/- SD, n=3 biological replicates. **c** Protein expression changes following *NRAS* knockdown in the stable cell lines. Representative images, n=2.

Figure 2c Uncropped western blots (some blots were cut in order to probe multiple antibodies)

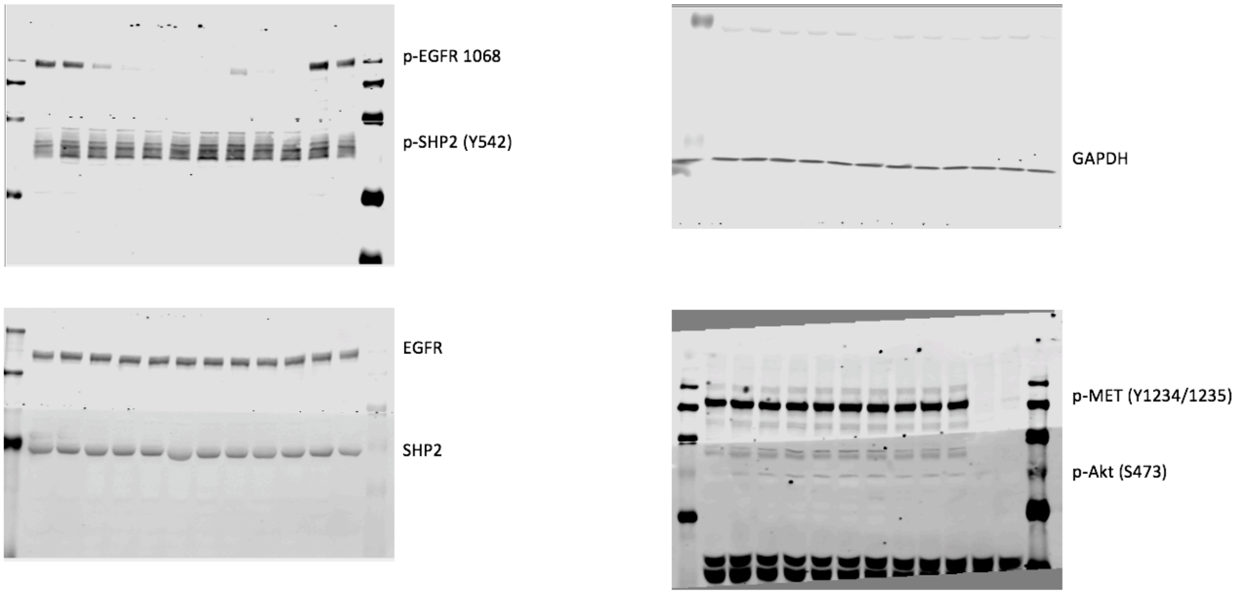

Figure 2c Uncropped western blots (some blots were cut in order to probe multiple antibodies)

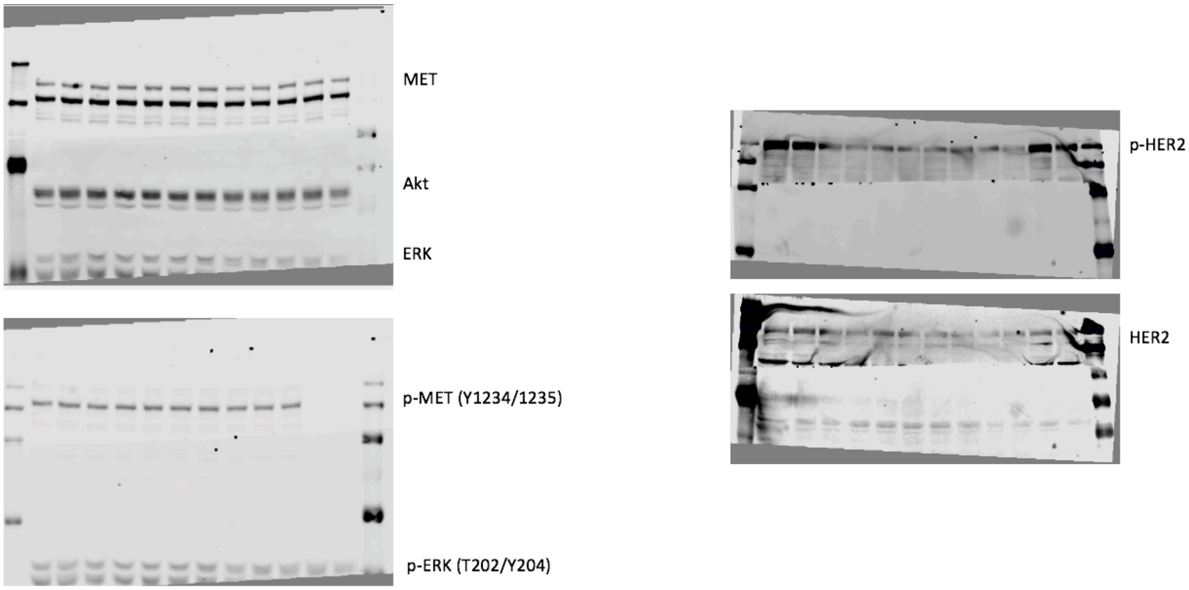

Figure 2f Uncropped westerns (some blots were cut in order to probe multiple antibodies)

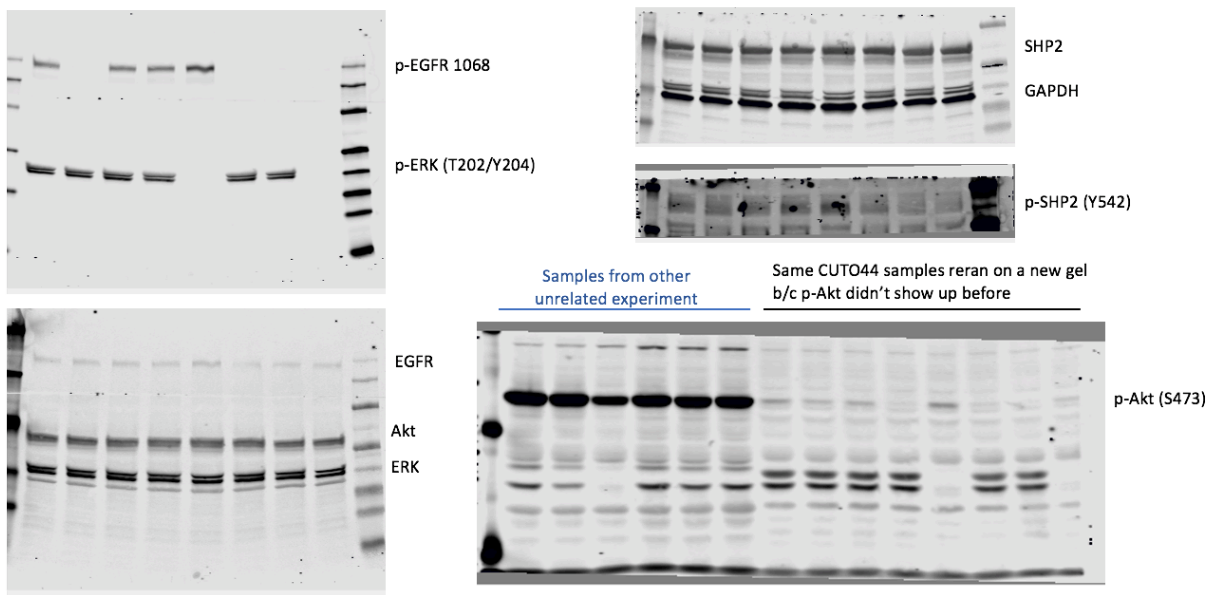

Figure 2f Uncropped western blots (some blots were cut in order to probe multiple antibodies)

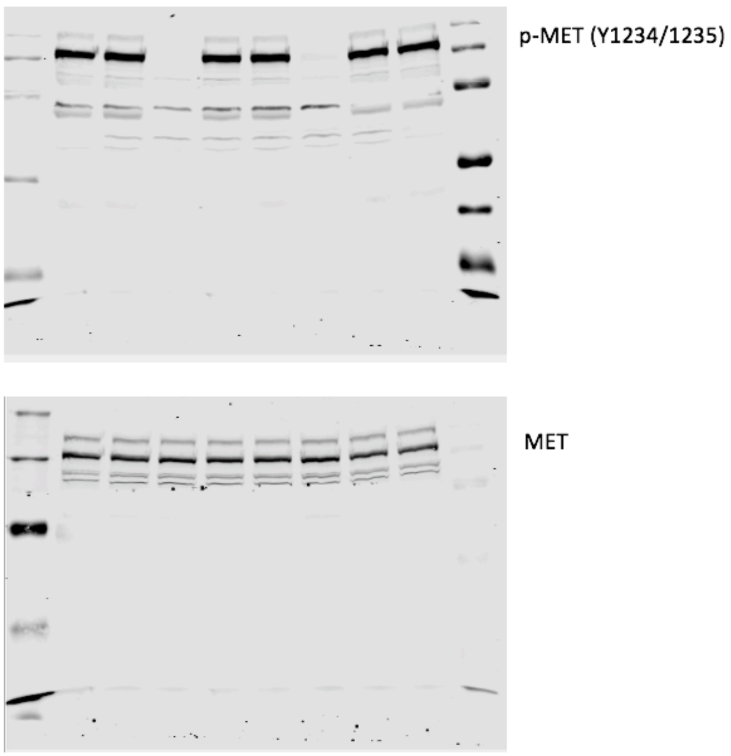

Figure 3b uncropped western blots (some blots were cut in order to probe multiple antibodies)

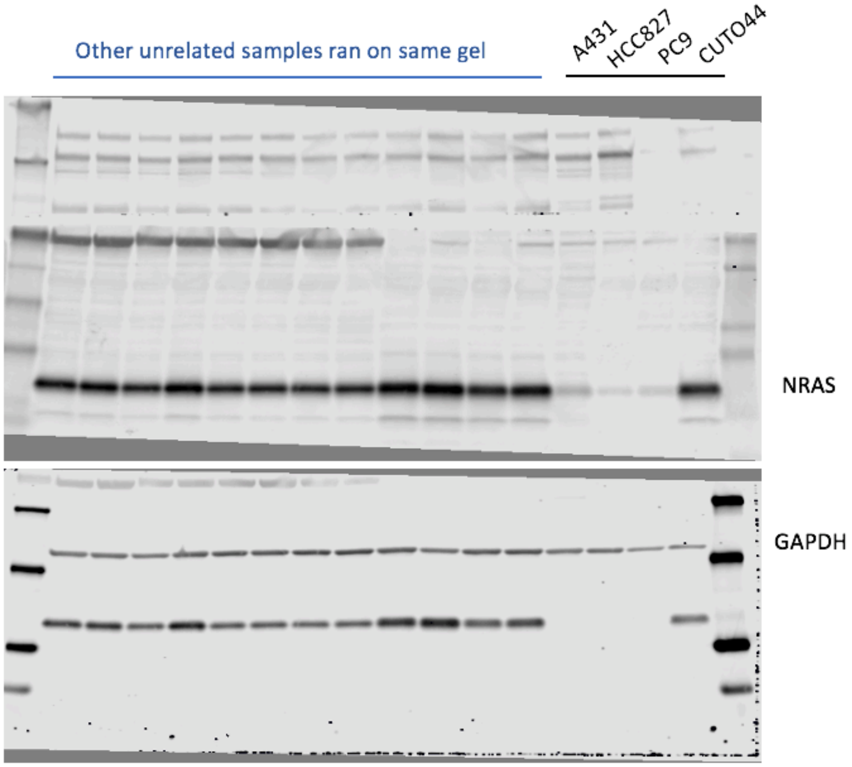

Figure 3d Uncropped western blots (some blots were cut in order to probe multiple antibodies)

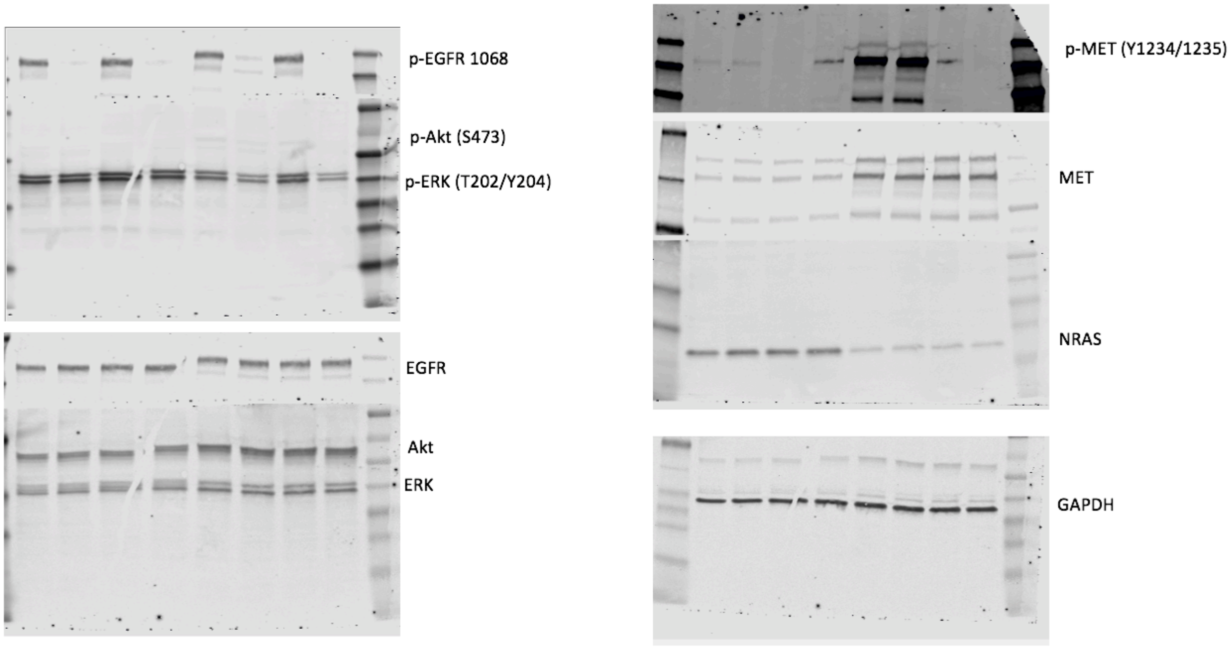

Supplementary Figure 3c uncropped western blots (some blots were cut in order to probe multiple antibodies)

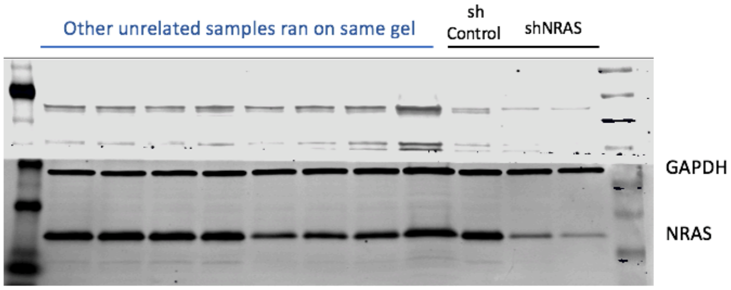

Supplement: Supplementary file 2 — Supplementary Information [file 41698_2021_231_MOESM2_ESM.pdf]
